# The relationships between clinical teaching behaviour and transition shock in newly graduated nurses

**DOI:** 10.1002/nop2.1458

**Published:** 2022-11-03

**Authors:** Bei Yun, Qian Su, Xuchun Ye, Yuhan Wu, Lian Chen, Yamei Zuo, Jia Liu, Lin Han

**Affiliations:** ^1^ School of Nursing Naval Medical University (Second Military Medical University) Shanghai China; ^2^ School of Nursing Lanzhou University Lanzhou China; ^3^ The First Clinical Medical College of Lanzhou University Lanzhou China; ^4^ Nursing Department Gansu Provincial Hospital Lanzhou China

**Keywords:** clinical teaching behaviour, newly graduated nurses, transition shock

## Abstract

**Aim:**

To explore the relationships between clinical teaching behaviour and transition shock in newly graduated nurses and significant differences in the northwest and northeast China.

**Design:**

A cross‐sectional design.

**Methods:**

A total of 211 (nurses) and 925 (preceptors) were recruited in six Third‐class different hospitals from July to August 2019.

**Results:**

The transition shock is negatively correlated with the clinical teaching behaviour in northwest and northeast China, while the transition shock in the northwest is higher than that in the northeast for the behindhand economy and natural limit.

**Conclusion:**

The transition process of newly graduated nurses in northeast China with ethnic minorities and the behindhand economy is more difficult. For newly graduated nurses, personal health and a supportive environment need further improvement. Teaching strategies for preceptors need to be enhanced. Two‐way feedback is more useful for both the newly graduated nurses and preceptors to improve quality care, and holistic care.

## 1 INTRODUCTION

World health organization in 2020 reported that there were 28 million nurses worldwide nowadays, though the number has increased by 4.7 million from 2013 to 2018. However, by 2020 the European Commission's report indicated there would be a shortage of 590,000 nurses worldwide (Zhang et al., 2017), and the number of nurses in the United States would reach 340,000. On average, one in six nurses will retire in the next 10 years as they grow older, among them, younger nurses might suffer from a higher turnover rate within their first year of professional healthcare actually (Ahlstedt et al., 2019). Specifically, the total number of registered nurses nationwide was about 4.45 million in 2020 in China, just 350,000 more than in 2018 (about 4.1 million). Nowadays, the shortage of nurses is very large, which seriously affects the level of medical care.

Generally, there are two reasons that result in the shortage of nurses, one is the relevant educated population is not large enough, leading to the potential nurse population cannot meet the demand. The other main reason is the high turnover rate of nurses, especially new nurses within their first year of professional healthcare, one of the prime origins is remarkable transition shock. Consequently, exploring an effective clinical teaching behaviour to comfort the transition shock of the nurses can help to reduce the high turnover rate, which makes a significant difference to healthcare over the world.

## 2 BACKGROUND

The transition shock refers to major challenges and difficulties that new nurses faced in their first year of employment during the transition from graduates to experienced nurses (Duchscher, 2009). The transition shock usually occurs in the transition from the theory learned in the academy to the clinical practice that is required in practice (Patterson et al., 2017) with feelings of anxiety and incompetence (Powers et al., 2019) in the first three months of employment (Zhang et al., 2017), which result from the patients’ workload, lack of nursing capacity and skills, and the gap between theory and practice (Labrague et al., 2018). During the transition process, the newly graduated nurses are expected to adapt to the new roles and bear more responsibilities to overcome the differences between the theoretical and the practical settings. Also, the newly graduated nurses need to integrate themselves into an unfamiliar environment, which emphasizes teamwork predominantly (Phillips et al., 2015). All these challenges and difficulties mentioned above make it difficult for the new nurses to adapt to the new work environment.

Higher transition shock results in a higher turnover rate of the new nurses, leading to the loss of the nurse population. A number of previous studies have discovered that a large proportion of newly graduated nurses experience a high turnover rate (Zhang et al., 2017). The average turnover rate in the health and social welfare sector is 3.0% (Suzuki et al., 2010), however, the turnover rate for newly graduated nurses in less than one year reached as high as 33.6% in South Korea in 2017 (Jung et al., 2019), while the turnover rate for newly graduated nurses in the first three months of employment reached a staggering 54.8%–57.7% in Taiwan (Lee et al., 2009). Thus, reducing the turnover rate significantly by comforting transition shock has been regarded as an efficient solution to solve the shortage of nurses.

Improving clinical teaching behaviour can comfort the transition shock of the nurses effectively, hence, the clinical preceptors play a dominant role in the process of supporting the nursing clinical experience practice. They can help new nurses establish a comfortable nursing environment (Lyman et al., 2020; Powers et al., 2019) by providing a work‐integrated learning opportunity, facilitating the nurses being socialized in the nursing department (Lee‐Hsieh et al., 2016), and emphasizing the importance of combining the clinical practice and nursing theory. All these activities promote the rapid development of clinical skills and nursing practice (Lovric et al., 2014; Voldbjerg et al., 2021). On the other hand, a previous systematic review indicated that clinical preceptors can play an important role in building the confidence of the newly registered nurses (Irwin et al., 2018), mitigating transition shock in the first year of new nurses in their professional transition period (Irwin et al., 2018; Powers et al., 2019; Rush et al., 2019).

However, a tremendous amount of effort is still needed to improve clinical teaching effectiveness for preceptors, because they are under great pressure on various teaching tasks and clinical work (Quek & Shorey, 2018). In practice, the absence of enough time and the imbalance between the working and teaching duties make the preceptors tired in the teaching process.

Therefore, clinical teaching is particularly important for the newly graduated nurses to go through the transition shock smoothly with the help of preceptors. However, previous clinical teaching has focused primarily on the preceptors or newly graduated nurses (Lee‐Hsieh et al., 2016) independently. There is an urgent need to build a systematic methodology that enables both the preceptors and the newly graduated nurses to evaluate their perceptions of clinical instruction in both directions. In our research, the Clinical Teaching Behaviour Inventory is a scale that can not only help preceptors to self‐evaluate according to their experience but also help newly graduated nurses to evaluate according to the preceptors’ teaching process.

Our research also has a wide range of practical implications and can be used as a reference for preceptors and newly graduated nurses all over the world, especially in economically underdeveloped countries and multi‐ethnic areas. Northeast China, with its developed economy, large population, and complete industrial categories, can represent a typical developing region. However, northwest China, with its behindhand economy and many ethnic minorities compared to northeast China, is a representative of an underdeveloped region in clinical practice. We made a contrast between these two regions in the relationship between the clinical teaching behaviour and transition shock.

In summary, our research established an effective way to study the potential relationships between clinical teaching behaviour and transition shock in newly graduated nurses and the significant differences between northwest and northeast China, which have stark economic disparities and minority conditions.

## 3 THE STUDY

### 3.1 Design

A quantitative, non‐experimental, cross‐sectional survey design with convenience sampling was adopted for the study where we collected 211 newly graduated nurses and 925 preceptors from July to August 2019 at six different Third‐class hospitals in the northeast and northwest China.

The newly graduated nurses included in this study possessed the following qualifications: (1) undergraduate diploma or above obtained from a technical secondary school, or junior college; (2) certified as a registered nurse; (3) working in a Third‐class hospital; (4) employed in the hospital after January 1, 2017. The preceptors included: (1) worked in Third‐class hospital hospitals; (2) certified as registered nurses; (3) worked in clinical teaching for more than 2 years; (4) volunteered to participate in the survey.

### 3.2 Questionnaires

#### 3.2.1 Demographic questionnaire

The demographic questionnaire included age, gender, education level, working time (years), classification of the department, employment type, whether served as a class cadre, whether the rotation was necessary, and the recruitment procedure used.

#### 3.2.2 The transition shock of Newly Graduated Nurses Scale

The scale evaluating transition shock was developed for the newly graduated nurses based on a previous study (Xue et al., 2015), which consisted of 27 items categorized into 4 dimensions: physical aspects, psychological aspects, knowledge and skills, social culture, and development. The scale scores varied from 27 to 135 after calculating the sum of the 4 dimensions and thereafter were clarified from 1 = completely disagree to 5 = completely agree on the 5‐Likert scale. The value of Cronbach's α coefficient was 0.918 and the content validity was 0.906. However, different items were included in each dimension of the scale to compare and analyse the scores of the scale. Score rate of the total score (or scores of each dimension)/full score of the scale (or full score of each dimension) × 100%.

#### 3.2.3 Clinical Teaching Behaviour Inventory

We investigated the clinical teaching behaviour of the preceptors by using the questionnaire developed by a Taiwan scholar in 2016, the Clinical Teaching Behaviour Inventory (Lee‐Hsieh et al., 2016). This scale contains 23 items that are evenly distributed over the following six distinct dimensions: Committing to teaching; building a learning atmosphere; using appropriate teaching strategies; guiding inter‐professional communication; providing feedback and evaluation; showing concern and support, which have can be considered as positive features. The response for each item was also graded on a 5‐point Likert scale: 1 = strongly disagree to 5 = strongly agree. The value of Cronbach's α was 0.96. The scale can be used for the preceptor's self‐evaluation and newly graduated nurses’ evaluation to potentially evaluate the clinical teaching behaviours related directly to their preceptor's clinical experience. The description of each version of the instrument was slightly different. For the preceptor self‐evaluation the subject of the scale was “I”, while for newly graduated nurses’ evaluation of the preceptor, it was the “preceptor”.

### 3.3 Data collection

The data was collected for this study from July to August 2019. First of all, we obtained consent from the hospital director of the nursing department, then, we selected a liaison officer at each hospital in order to enable them to understand the purpose and meaning of this study. Thereafter, liaisons selected newly graduated nurses and preceptors who have met inclusion as well as exclusion criteria and sent electronic questionnaires to the newly graduated nurses and preceptors by an APP called “Wenjuanxing”. By using this APP, the questionnaires can be filled out online, which took at least 5 minutes, and the researchers could observe the results afterward. At last, the researchers reviewed the completion level as well as completion time, and then some missing data and data with a short completion time were deleted to guarantee the quality of the data. The findings included 205 newly graduated nurses’ questionnaires and 910 preceptors’ questionnaires after eliminating 6 and 15 unqualified questionnaires, the effective recovery was found to be 97.16% and 98.38%, respectively.

### 3.4 Data analysis

The study data were analysed using the SPSS 25.0 package program. Initially, descriptive statistics, such as the percentage, arithmetic mean, and standard deviation were used in the analysis of sociodemographic characteristics, the Clinical Teaching Behaviour Inventory, and The Transition Shock of New Graduated Nurses Scale in northeast and northwest China. Thereafter, One‐Way ANOVA and Independent sample t‐test were used to explore the potential impact of demographic characteristics on transition shock for newly graduated nurses and to explore the impact of transition shock for new nurses in different regions. In order to identify specific characteristics linked to transition shock and clinical teaching behaviour for newly graduated nurses in northeast and northwest China, bivariate analyses were conducted by using Pearson correlations. A multivariate stepwise regression analysis was then conducted to explore the influencing factors of transition shock for the newly graduated nurses in northwest and northeast China. The statistical significance was set at *p* < .05.

### 3.5 Validity and reliability

The questionnaires have been validated in previous research. The Cronbach's α coefficient of Transition Shock of Newly Graduated Nurses Scale was 0.918 and the content validity was 0.906, and the Clinical Teaching Behaviour Inventory showed a Cronbach's α of 0.96.

### 3.6 Ethical consideration

This study was approved by Medical Ethics Committee, School of Nursing, Lanzhou University (No. HLLL20191218). Besides, In the invitation letter, respondents were informed about the purpose of the study, the voluntary nature of completing submitting the questionnaire, and that the data would be handled without identifying information and only by members of the research group. Submitting the questionnaire was considered as consent to participate in the study.

## 4 RESULTS

### 4.1 Participants

The majority of the newly graduated nurses between 21–25 years old were female with a percentage of 95.7% in the northwest and 94.3% in the northeast. In the northwest, the nurses possessed bachelor's degrees (93.2%), while in the northeast, the number of junior colleges (48.9%) and bachelor's newly graduated nurses (50%) were nearly equal, Please see Table 1 for details. There were no statistically significant differences in all the demographic characteristics on the transition shock (*p >* .05), except for the working time in the northwest (*p* < .05).

**TABLE 1:** Demographic characteristics and transition shock for the newly graduated nurses

	*N*		Mean (SD)		*F* /*t*		*p*	
	NW	NE	NW	NE	NW	NE	NW	NE
*Age*								
21–25	81 (69.2)	50 (56.8)	86.90 (17.06)	75.36 (25.10)	2.253	0.210	0.110	0.811
26–30	33 (28.2)	30 (34.1)	94.24 (18.52)	72.67 (22.46)				
≥30	3 (2.6)	8 (9.1)	94.67 (5.13)	78.38 (28.67)				
*Gender*								
Male	5 (4.3)	5 (5.7)	88.00 (11.34)	57.60 (21.87)	−0.152	−1.635	0.880	0.106
Female	112 (95.7)	83 (94.3)	89.22 (17.80)	75.75 (24.21)				
*Education level*								
Junior college	5 (4.2)	43 (48.9)	92.00 (16.64)	74.72 (25.55)	0.228	0.638	0.796	0.531
Bachelor	109 (93.2)	44 (50)	89.20 (17.56)	74.09 (23.30)				
Master or above	3 (2.6)	1 (1.1)	83.33 (13.58)	102.00 (0)				
*Whether served as a class cadre*								
Yes	49 (41.9)	41 (46.6)	90.29 (15.11)	75.51 (24.34)	0.582	0.285	0.562	0.776
No	68 (58.1)	47 (53.4)	83.37 (19.17)	74.02 (24.57)				
*Working time (years)*								
3	40 (34.2)	12 (13.6)	93.93 (18.93)	84.58 (2.82)	6.243	1.150	0.003	0.322
2	54 (46.1)	3 (3.4)	90.15 (16.28)	72.00 (37.24)				
1	23 (19.7)	73 (83)	78.61 (13.71)	73.21 (24.05)				
*Classification of department*								
Surgical	38 (32.5)	23 (26.2)	85.95 (16.48)	70.52 (24.44)	0.700	0.344	0.554	0.794
Internal medicine	45 (38.5)	48 (54.5)	91.42 (19.63)	75.58 (23.32)				
Emergency	14 (12)	5 (5.7)	89.00 (18.80)	79.00 (34.89)				
Intensive care units	20 (17)	12 (13.6)	90.35 (13.42)	77.50 (25.80)				
*Whether the rotation is necessary*								
Very necessary	27 (23.1)	35 (39.8)	91.19 (22.38)	77.26 (29.21)	0.507	0.911	0.678	0.439
Be necessary	79 (67.5)	46 (52.3)	88.59 (16.22)	73.28 (20.98)				
Unnecessary	9 (7.7)	6 (6.8)	85.78 (13.45)	76.83 (14.69)				
Very unnecessary	2 (1.7)	1 (1.1)	100.00 (14.14)	39.00 (0)				
*Recruitment method*								
Formal employee	3 (2.6)	8 (9.1)	74.00 (19.98)	84.63 (30.68)	1.338	1.028	0.267	0.362
Informal employee	109 (93.2)	5 (5.7)	89.78 (17.34)	65.60 (20.10)				
Others	5 (4.2)	75 (85.2)	85.00 (20.01)	74.27 (23.85)				

Abbreviations: NE, in the northeast; NW, in the northwest.

### 4.2 The Transition Shock of New Graduated Nurses Scale

The mean scores for the transition shock were M = 89.17, SD = 17.54 in the northwest, and M = 74.72, SD = 24.33 in the northeast. The dimensions were listed from high to low and included: Physical aspect; knowledge and skills, psychological aspect, and social and development. The impact of the transition of new nurses in different regions is statistically significant (*p* < .05) (Table 2).

**TABLE 2:** The level of the transition shock and its differences in the different areas of China

	Sum (M(SD))		%		*t*
	NW	NE	NW	NE	
PHY	22.08 (4.54)	19.77 (6.55)	73.6	65.9	2.828*
PSY	26.57 (6.47)	22.53 (8.28)	66.43	56.33	3.786*
KT	16.89 (3.79)	14.27 (5.27)	67.56	57.08	3.952*
SO	23.63 (7.03)	18.14 (7.87)	59.08	45.35	5.261*
TS	89.17 (17.54)	74.72 (24.33)	66.05	55.35	4.725*

Abbreviations: KT, knowledge and skills; NE, in the northeast; NW, in the northwest; PHY, physical aspects; PSY, psychological aspects; SO, social culture and development; TS, Transition shock.

* *p* < .01.

### 4.3 Clinical Teaching Behaviour Inventory

**FIGURE 1:**
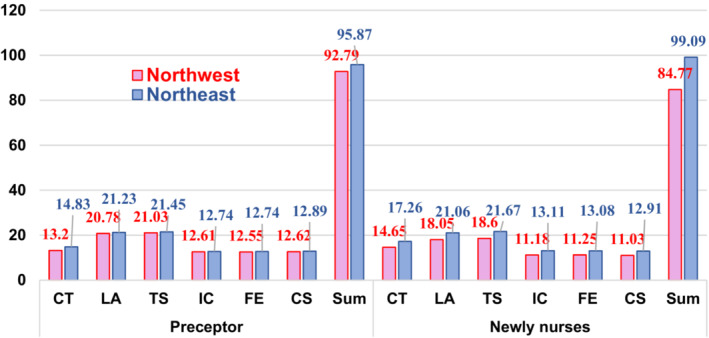
A comparison of the mean score of the clinical teaching behaviour for the preceptors and new nurses in both the regions. CS, showing concern and support; CT, committing to teaching; FE, providing feedback and evaluation; IC, guiding inter‐professional communication; LA, building a learning atmosphere; TS, using appropriate teaching strategies.

The mean scores for the clinical teaching behaviour were M = 92.79, SD = 11.57, and M = 95.87, SD = 10.92 for preceptors, M = 84.77, SD = 14.21, and M = 99.09, SD = 13.85 for the newly graduated nurses in the northwest and northeast China, respectively. In addition, the mean scores of the preceptor's self‐evaluation and newly graduated nurses’ evaluation of clinical teaching behaviour have been shown in Figure 1. It was also observed that regardless of who was evaluated, the clinical teaching behaviour in the northeast was significantly higher than in the northwest (see Figure 1).

For preceptors’ self‐evaluation, in the northwest and northeast, the various dimensions were indicated from high to low and included: showing concern and support; using appropriate teaching strategies; guiding inter‐professional communication; providing feedback and evaluation; building a learning atmosphere; and committing to teaching.

For the evaluation of the newly graduated nurses in the northwest, the dimensions were listed from high to low which included: providing feedback and evaluation; guiding inter‐professional communication; using appropriate teaching strategies; showing concern and support; committing to teaching, and building a learning atmosphere. While in the northeast, the dimensions were listed from high to low and included: Guiding inter‐professional communication; providing feedback and evaluation; using appropriate teaching strategies; Committing to teaching; showing concern and support, and building a learning atmosphere (Figure 1).

### 4.4 Correlation between the newly graduated nurses’ transition shock and clinical teaching behaviour

Pearson correlations between the studied variables have been shown in Table 3. In the northwest, the transition shock had negative correlations with clinical teaching behaviour (*r* = −0.335, *p* < .001). Transition shock displayed the highest negative correlation with providing feedback and evaluation (*r* = −0.369, *p* < .001), followed by showing concern and support (*r* = −0.331, *p* < .001), while had the lowest negative correlation with Committing to teaching (*r* = −0.252, *p* < .001). In the northeast, transition shock displayed a negative correlation with Committing to teaching (*r* = −0.243, *p* < .05).

**TABLE 3:** The potential correlations between the transition shock and clinical teaching Behaviour

	TS	PHY	PSA	KT	SO
*NW*					
CTBI	−0.335**	−0.248**	−0.284**	−0.117	−0.350**
CT	−0.252**	−0.133	−0.201*	−0.108	−0.301**
LA	−0.284**	−0.197*	−0.263**	−0.086	−0.292**
TS	−0.318**	−0.252**	−0.287**	−0.079	−0.323**
IC	−0.300**	−0.243**	−0.239**	−0.092	−0.322**
FE	−0.369**	−0.303**	−0.304**	−0.177	−0.349**
CS	−0.331**	−0.266**	−0.260**	−0.126	−0.348**
*NE*					
CTBI	−0.173	0.000	−0.236*	−0.104	−0.217*
CT	−0.243*	−0.108	−0.279**	−0.165	−0.257*
LA	−0.152	0.029	−0.234*	−0.067	−0.203
TS	−0.159	−0.011	−0.210*	−0.097	−0.195
IC	−0.139	0.044	−0.200	−0.099	−0.190
FE	−0.112	0.050	−0.181	−0.063	−0.155
CS	−0.158	0.026	−0.211*	−0.099	−0.223*

Abbreviations: CS, showing concern and support; CT, Committing to teaching; CTBI, clinical teaching behavior; FE, providing feedback and evaluation; IC, guiding inter‐professional communication; LA, building a learning atmosphere; NE, in the northeast; NN, newly graduated nurses; NW, in the northwest; P, preceptors; TS, using appropriate teaching strategies.

* *p* < .05;

** *p* < .01.

### 4.5 Factors associated with the transition shock of newly graduated nurses

In the northwest, six different dimensions of scales and education levels of preceptors, newly graduated nurses’ age, and working time were considered as independent variables, whereas transition shock was seen as dependent variables, and the stepwise regression analysis showed that *F* = 11.256, *p* = .000, thereby indicating that the regression equation was statistically significant. The variables entering the regression model included working time, education level of preceptors, and providing feedback and evaluation accounting for 82.07% of the total variation in the newly graduated nurses’ transition shock.

In the northeast, the six dimensions of the clinical teaching behaviour were found to be independent variables, transition shock was seen as the dependent variable, and the stepwise regression analysis showed that *F* = 5.405, *p* = .022, thus indicating that the regression equation was statistically significant. The variables entering the regression model included committing to teaching which accounted for 30.46% of the total variation in the newly graduated nurses’ transition shock (Table 4).

**TABLE 4:** Multiple regression analysis associated with the various influencing factors of transition shock across the newly graduated nurses

	Regression coefficient	Standard error	Standardized regression coefficient	*t*‐value	*p*‐value
*NW*					
Constant	158.010	14.033		11.260	<0.001
FE	−2.802	0.729	−0.322	−3.845	<0.001
Working time	−5.752	2.028	−0.237	−2.836	0.005
Educational level	−9.338	4.102	−0.188	−2.277	0.025
*NE*					
Constant	114.447	17.276		6.625	<0.001
CT	−2.302	0.990	−0.243	−2.325	0.022

Abbreviations: CT, committing to teaching; FE, providing feedback and evaluation; NE, in the northeast; NW, in the northwest.

## 5 DISCUSSION

As an important mainstream force in the future of the nursing workforce, it is necessary to understand the extent of the transition from students to nurses, and the various factors that can potentially affect the transformation of the newly graduated nurses. The present study provided evidence for the different predictors of the impact of the newly graduated nurse's transformation in the course of their work, supportive environment, and culture, as well as provided feedback and evaluation by preceptors, standardized training for the preceptors, and so on.

### 5.1 The impact of the transition shock for the newly graduated nurses

#### 5.1.1 The level of the transition shock

Following the results, the study showed that the scoring rate of the transition shock of newly graduated nurses was 66.1% in the northwest and 55.3% in the northeast, which was relatively low compared with those of the developed cities in China. For instance, in Fujian, the scoring rate of the transition shock of newly graduated nurses was 62.6% (Zeng et al., 2021); in Shanghai, the scoring rate of transition shock of the newly graduated nurses was 76.8% (Zhang et al., 2017), but the scoring rate was 61% in the northeast China (Chen et al., 2021), which was similar to the result of the present studies. The hospitals evaluated in this study are all located in underdeveloped areas in China, compared with Shanghai, one of the largest cities in mainland China, and their standards and entry requirements were lower than those in developed areas. Therefore, the impact of transformation was at a moderate level.

#### 5.1.2 The dimension of the physical aspect

In this study, the physical aspect, knowledge and skills, and psychological aspect are the three top dimensions that might have a great impact on the results, which was in parallel to the findings of the prior studies (F. Chen et al., 2021). A detailed discussion has been indicated below.

The physical aspect has been considered the highest scoring dimension, thus indicating the physical aspect is the most important. The workload of the newly graduated nurses is generally large and heavy task is often assigned to them, which causes them to prolong the working time to accomplish their assigned tasks. Furthermore, frequent nursing shifts for newly graduated nurses can lead to serious adverse effects and can result in physical fatigue, back pain, sleep disorders, and other symptoms. Moreover, night shift nurses often work alone, which can put them under greater pressure (Chen et al., 2014; Fu et al., 2018), and make them vulnerable to physical and psychological exhaustion (de Cordova et al., 2016; Kwak et al., 2018; Nowrouzi et al., 2016). Besides, it can be difficult for shift nurses to balance their work, family, and social life (Chen et al., 2014; de Cordova et al., 2016).

Moreover, because most nurses in China possess junior college degrees, undergraduate students have gradually become the mainstream source of new nurses, which has prompted the head nurse and nursing managers to pay more attention and provide better training to undergraduate students. New undergraduate nurses must not only complete daily nursing work but also are expected to continue their education, scientific research, etc., thus making them even more exhausted. Consequently, the head nurse should arrange work hours reasonably so that the actual ability of the new nurse can effectively match the workload and give the newly graduated nurses sufficient time to familiarize and understand the department in a gradual manner.

#### 5.1.3 The dimension of knowledge and skills

Knowledge and skills were found to be the second‐highest dimension, which might contribute to the performance of newly graduated nurses who were unable to perform their job duties during the transition period due to their limitations associated with a lack of professional knowledge and skills that might not be able to meet the requirements of actual work (Duchscher, 2009). Newly graduated nurses indicated that it was difficult to decrease the gap between theoretical knowledge and clinical practice and adjust effectively to their role as nurses and become a part of the nursing department's group (Powers et al., 2019; Ward & McComb, 2017). Moreover, newly graduated nurses have only a few opportunities to simulate the real clinical environment before they enter the nursing practice so they have hardly reinforced their clinical reasoning and critical thinking in practical settings. What's more, their textbook knowledge was found to be lagging, thus making newly graduated nurses constantly apply outdated knowledge that made it difficult for them to adapt to the changing clinical practices. Besides, during the transition process, newly graduated nurses have been found to be prone to nursing adverse events due to a lack of adequate nursing skills and complete knowledge about their field (Murray et al., 2019). Therefore, newly graduated nurses have to cope with the stress related to the complexity of practice, and also the gap between academic knowledge and clinical practice to markedly decrease the rate of nursing adverse events (Hayes, 2018; Treiber & Jones, 2018).

#### 5.1.4 The dimension of the psychological aspect

The psychological aspect was observed to be the third‐highest dimension. When nursing students are in the internship phase, the teacher would assign them, stable patients, for nursing; but when they enter the clinical work, newly graduated nurses need to accept patients with complicated conditions immediately and assume several daily responsibilities, including those related to documentation, answering calls, inter‐professional communication, and so on, which have been found to be the primary reasons for the transition shock (Powers et al., 2019). Furthermore, the imagination of newly graduated nurses is often unrealistic, so they do not have enough preparation to face the actual clinical practice due to the differences between learning experiences during education and the realities associated with the profession (Dwyer & Hunter Revell, 2016; El Haddad et al., 2017; Walker et al., 2017). They may face role stress, psychological stress (Kaihlanen et al., 2020), and even a decline in quality (Hasson & Gustavsson, 2010). Consequently, head nurses should create a conducive atmosphere of learning, mutual care, and help, thus providing the newly graduated nurses with enough encouragement and two‐way feedback.

### 5.2 The impact of the clinical teaching behaviour

#### 5.2.1 The newly graduated nurses and preceptors evaluation of clinical teaching behaviour

The scoring rate for dimensions of clinical teaching behaviour from high to low has been shown in Table 3. Based on the two‐way assessment, preceptors and the newly graduated nurses all thought that building a learning atmosphere and committing to teaching needed to be improved and strengthened by creating a safer environment and trusting atmosphere (Bott et al., 2011; Lazarus, 2016; Murray et al., 2019). However, due to the heavy workload in the nursing department, preceptors also might not find enough time to communicate effectively with the newly graduated nurses and so it can be difficult to build a good and supportive atmosphere (Barker & Pittman, 2010; Carlson et al., 2010).

Besides, although preceptors are specialists in the nursing profession and do well in dealing with the complex emergency, they often do not experience standard and systematic training in clinical teaching and are thus unable to impart their practical experiences to the newly graduated nurses (Cline et al., 2017), and a previous study has shown that it was also necessary for the preceptors to participate in regular training exercises (Horton et al., 2012). Only when preceptors’ teaching methods are consistent with that of the newly graduated nurses’ learning methods, they can provide a good balance of teaching as well as learning, which can facilitate the newly graduated nurses to learn more effectively (O'Mahony et al., 2016). However, due to the poor balance between the nursing work and nursing teaching tasks, preceptors do not have enough time to explore the targeted teaching for each new nurse. As a result, building a learning atmosphere and committing to teaching are the most important dimensions that have to be improved substantially for the newly graduated nurses to overcome transition shock (Zhang et al., 2017).

Moreover, based on the newly graduated nurses’ evaluation, providing feedback and evaluation was the highest dimension, which was also consistent with the dimension of showing concern and support for preceptors’ self‐evaluation. Providing constructive feedback, no matter positive or negative could be useful for the newly graduated nurses and the preceptors to improve their critical thinking (Lazarus, 2016), which is the key aspect of the clinical education experience and important for the development of a competent clinical preceptor (Buck et al., 2014; Wilkinson et al., 2013). Newly graduated nurses are frequently involved in complex and busy situations whereas the newly graduated nurses are required to acquire the capability of solving problems and making decisions.

However, during this process, preceptors should show concern and support for the newly graduated nurse's work so that newly graduated nurses could correct their own mistakes. Moreover, based on the mistakes made by these nurses, preceptors can analyse the factors that led to these mistakes, design measures to correct these mistakes and explain the right procedures to newly graduated nurses, which can effectively improve and stimulate the preceptors’ critical thinking. Besides, the newly graduated nurses often take preceptors’ guidance and feedback seriously when they enter a new environment, so the mechanism of providing feedback and evaluation was found among the highest dimensions. Furthermore, for the preceptors, the daily guidance and instructions for the newly graduated nurse should involve their care and encouragement.

#### 5.2.2 The impact of the transition shock and clinical teaching behaviour on different regions

As shown in the results, the transition shock in the northwest was significantly higher than that in the northeast. It is possible that owing to different educational levels, high‐degree nurses were unable to adapt to true clinical nursing work and even could not accept themselves doing some basic nursing work. However, there were more bachelor's degree holders among the newly graduated nurses, in the northwest than in the northeast. Furthermore, the number of Third‐class hospitals in the northwest is less than that in the northeast, which has led to great competition for employment among the newly graduated nurses, who also suffer from job insecurity that they might be fired for not doing well in the work process. In addition, due to the many ethnic minorities in northwest China, the number of ethnic minority patients in Third‐class hospitals was relatively large, which might have increased the pressure of nursing work for the newly graduated nurses to some extent. For example, poor verbal communication leads to the inaccurate transmission of nursing knowledge, differences in religious beliefs make it difficult to implement some of the care measures, some entrenched lifestyle habits are detrimental to health, and other various reasons can make the newly graduated nurses face various challenges when performing their nursing operations. As a consequence, transition shock in the northwest was markedly higher than that in the northeast.

It was found that irrespective of the preceptors’ or the newly graduated nurses’ evaluation, clinical teaching behaviour in the northwest was significantly lower than in the northeast. Above all, compared with northeast China, northwest China is in inland China, which might contribute to the newly graduated nurses and preceptors possessing limited thinking and weak ability to accept new knowledge, so the newly graduated nurses were unable to cope with clinical nursing work, whereas the preceptors always focused only on their nursing job and did not pay much attention to the teaching tasks. Therefore, the development of nursing education in northwest China was relatively slow compared with that in northeast China (Table 1), so the education level of preceptors was found to be generally lower, resulting in the teaching level of the preceptors not being sufficient to meet the clinical teaching requirements of the new nurse. Moreover, since there are fewer universities in northwest China than in northeast China, many preceptors have not received standardized training from reputed universities, so their clinical teaching behaviours were inadequate as compared to those in northeast China.

### 5.3 The relationships between clinical teaching behaviour and transition shock

Transition shock has been negatively correlated with clinical teaching behaviour, which indicates that to make the newly graduated nurses experience the transition period successfully and retain them, the improvement of clinical teaching behaviour is very important. Preceptors, as an important part of the clinical nursing teaching, not only carry out their own duties in the course of work but must also take responsibility for the teaching of new nurses (Quek & Shorey, 2018). While teaching newly registered nurses about the clinical practice, their passion for nursing and attitude toward the patients have a profound impact on the future work of new nurses (Voldbjerg et al., 2021). A qualitative, phenomenological study (Wildermuth et al., 2020) revealed that the positive communication between the newly graduated nurse and the preceptor, as well as the continuous positive feedback and support from the preceptor, were extremely pivotal for the newly graduated nurses to go through the transition smoothly, which was in parallel to the findings of the prior studies (Murray et al., 2019).

According to the aforementioned discussion and the study findings, the directors of nursing and head nurses should explore optimal clinical teaching methods, clinical teaching models, and teaching facilities to significantly reduce the transition shock for newly graduated nurses. At first, the head nurses could take appropriate actions from the view of the balance between life and work. It can be concluded from our findings that by providing flexible scheduling, reasonable workloads, and sufficient staffing through the nurse's personal situation, the head nurses can effectively help the nurses to achieve proper work‐life balance to mitigate transition shock. In addition, hospitals should standardize the teaching and learning procedures. Firstly, hospital leaders should standardize the selection criteria for the qualifications of the teachers in order to improve the teaching staff quality. Secondly, the directors of nursing should screen out head nurses with strong core competence skills to create a good department environment and culture, which can be extremely crucial for the nurses to stay in the department for a long time. Last but not least, two‐way feedback mechanism between the teachers and students can also be effective in improving the abilities of both parties. Finally, universities and clinical facilities need cooperation to help to bridge the gap between theoretical and practical knowledge. They can cooperate to hold high‐simulation simulation teaching to improve the student's critical thinking and produce advanced beginners, rather than novice nurses, which can prove to be effective to ease the transition process for the newly graduated nurses.

## 6 LIMITATIONS

The study has several limitations. Firstly, this study adopted a convenient sampling method, which might have led to a large deviation in results. Secondly, because of the relatively small, recruited number of participants, our study may have limited generalizability and more research with larger samples is required to confirm our findings. Thirdly, the cross‐sectional design of our current study allowed only a temporal snapshot of the transition shock at a given time. Thus, the panel data collected for this population may give a more precise estimation of the various key factors associated with the transition shock.

## 7 CONCLUSION/IMPLICATIONS FOR PRACTICE

This study is the first to examine the potential relationship between the clinical teaching behaviour of the newly graduated nurses in Northeast and Northwest China and the impact of transition shock. The findings indicated that due to a large number of ethnic minorities and underdeveloped factors in northwest China, the transition process of newly graduated nurses in its region is more difficult and faces a greater impact than that of newly graduated nurses in northeast China. Besides, whether in the northwest or northeast of China, it was important for the newly graduated nurses to improve their clinical teaching behaviour to go through the transition shock successfully. It was also found that transition shock had a negative effect on clinical teaching. The study showed that attention should be paid to the physical aspect of the newly graduated nurses, as well as measures that should be adopted for establishing a supportive environment and culture for the nursing department, and the teaching strategies for preceptors. In addition, the study suggested that two‐way feedback was also very important for both the newly graduated nurses and preceptors. Overall, the implementation of measures to solve these problems can significantly improve quality care, and holistic care, and reduce the pressure on the newly graduated nurses.

## AUTHOR CONTRIBUTIONS

Study conception and design: Lin Han and Xuchun Ye. Data collection: Lin Han, Bei Yun, Yamei Zuo and Jia Liu. Data analysis and interpretation: Bei Yun, Qian Su, Yuhan Wu and Lian Chen. Drafting of the article: Bei Yun. Critical revision of the article: Lin Han and Xuchun Ye.

## FUNDING INFORMATION

This project was supported by Scientific Research Project of health industry in Gansu Province (grant number: GSWSKY‐2019‐41); the Fundamental Research Funds for the Central Universities (grant numbers: lzujbky‐2016‐ct14; lzujbky‐2018‐ct05).

## CONFLICT OF INTEREST

The authors declare no conflicts of interest.

## ETHICAL APPROVAL

This study was approved by Medical Ethics Committee, School of Nursing, Lanzhou University (No. HLLL20191218).
